# Data Resource Profile: The HUNT Biobank

**DOI:** 10.1093/ije/dyae073

**Published:** 2024-06-04

**Authors:** Marit Næss, Kirsti Kvaløy, Elin P Sørgjerd, Kristin S Sætermo, Lise Norøy, Ann Helen Røstad, Nina Hammer, Trine Govasli Altø, Anne Jorunn Vikdal, Kristian Hveem

**Affiliations:** HUNT Research Centre, Department of Public Health and Nursing, Faculty of Medicine and Health Sciences, Norwegian University of Science and Technology, Trondheim, Norway; Levanger Hospital, Nord-Trøndelag Hospital Trust, Levanger, Norway; HUNT Research Centre, Department of Public Health and Nursing, Faculty of Medicine and Health Sciences, Norwegian University of Science and Technology, Trondheim, Norway; Levanger Hospital, Nord-Trøndelag Hospital Trust, Levanger, Norway; Department of Community Medicine, Center for Sami Health Research, Arctic University of Norway, Tromso, Norway; HUNT Research Centre, Department of Public Health and Nursing, Faculty of Medicine and Health Sciences, Norwegian University of Science and Technology, Trondheim, Norway; Levanger Hospital, Nord-Trøndelag Hospital Trust, Levanger, Norway; HUNT Research Centre, Department of Public Health and Nursing, Faculty of Medicine and Health Sciences, Norwegian University of Science and Technology, Trondheim, Norway; Levanger Hospital, Nord-Trøndelag Hospital Trust, Levanger, Norway; HUNT Research Centre, Department of Public Health and Nursing, Faculty of Medicine and Health Sciences, Norwegian University of Science and Technology, Trondheim, Norway; Levanger Hospital, Nord-Trøndelag Hospital Trust, Levanger, Norway; HUNT Research Centre, Department of Public Health and Nursing, Faculty of Medicine and Health Sciences, Norwegian University of Science and Technology, Trondheim, Norway; Levanger Hospital, Nord-Trøndelag Hospital Trust, Levanger, Norway; HUNT Research Centre, Department of Public Health and Nursing, Faculty of Medicine and Health Sciences, Norwegian University of Science and Technology, Trondheim, Norway; Levanger Hospital, Nord-Trøndelag Hospital Trust, Levanger, Norway; HUNT Research Centre, Department of Public Health and Nursing, Faculty of Medicine and Health Sciences, Norwegian University of Science and Technology, Trondheim, Norway; Levanger Hospital, Nord-Trøndelag Hospital Trust, Levanger, Norway; HUNT Research Centre, Department of Public Health and Nursing, Faculty of Medicine and Health Sciences, Norwegian University of Science and Technology, Trondheim, Norway; Levanger Hospital, Nord-Trøndelag Hospital Trust, Levanger, Norway; HUNT Research Centre, Department of Public Health and Nursing, Faculty of Medicine and Health Sciences, Norwegian University of Science and Technology, Trondheim, Norway; HUNT Center for Molecular and Clinical Epidemiology, Department of Public Health and Nursing, Faculty of Medicine and Health Sciences, Norwegian University of Science and Technology), Trondheim, Norway; Department of Research, St Olav’s Hospital, Trondheim, Norway

Key Features

- The Trøndelag Health Study (HUNT) Biobank, located at Levanger, Mid-Norway, holds biological material from 110 000 participants (aged 13–100 years) from the longitudinal population-based HUNT Study, Norway, including whole blood, serum, plasma, DNA, RNA, urine, saliva and fecal samples, optimally handled and stored for research purposes.
- Among 94 000 participants with biological material, repeated measurements and a wide collection of processed samples are available from 26 000 that have participated in three successive HUNT surveys (HUNT2–4, 1995–2008).
- Large-scale, multi-omics analyses based on the existing sample collections are available.
- A unique personal identification number (PIN) for all citizens enables linkage to a wide range of local and national registries containing numerous incident clinical endpoints.
- Data and biological materials are accessible on request for national and international research groups, which also includes industrial-based research collaborations.

## Data resource basics

The Trøndelag Health Study (HUNT) is a large, population-based, longitudinal health study in central Norway, consisting of four surveys conducted in 1984–86 (HUNT1), 1995–97 (HUNT2), 2006–08 (HUNT3) and 2017–19 (HUNT4).^1–3^ Participation rates were 89%, 69%, 54% and 54%, respectively (see participation flowchart in Supplementary Figure S1, available as Supplementary data at *IJE* online). The study was initially established to study the prevalence of hypertension, diabetes and general health in a large population cohort, and presently HUNT includes data from 250 000 participants aged 13 years and above, 110 000 with biological material. Biological material has been collected through screening sites in all municipalities of the northern part of Trøndelag County. All clinical examinations have been performed in temporarily established screening sites.^2^ Funding and ethical clearance are described in more detail below. Approximately 26  000 individuals have participated in three successive HUNT surveys (HUNT2–4, 1995–2008) with repeated measurements and new collections of biological samples.

A resent follow-up of the HUNT4 Survey, the HUNT COVID Study (2021–23), has just been completed, focusing on the impact of the COVID-19 pandemic and of its accompanying nationwide control measures on public health. HUNT5 is in the planning phase and will be conducted in 2027–29.

The Young-HUNT Study, also part of the HUNT Study, consists of four surveys, Young-HUNT1 (1995–97), Young-HUNT2 (2000–01), Young-HUNT3 (2006–08) and Young-HUNT4 (2017–19), including all junior and senior high school students (aged 13–19 years) in the county with participation rates of 76–95%.^4^^,^^5^ DNA is collected from buccal swabs and saliva from the Young-HUNT3 and Young-HUNT4 participants.

The data resource is based on collected questionnaires, interviews, clinical examinations and biological material. A unique personal identification number (PIN) enables linkage to hospital records and local and national registries, adding significant value to the data source. The extensive number of participants, the samples collected, available analyses and longitudinal study design with 40 years of follow-up, have made the HUNT Study a highly attractive data resource for all types of epidemiological studies, disease risk prediction, early biomarker detection and clinical translation. It offers a unique platform for both association and prospective follow-up studies. Through cutting-edge integrative studies, it is possible to explore the polygenic architecture of complex diseases and study the impact of functional genetic variants and gene-environment interactions on various health outcomes. The extensive health data resource in combination with multiple genome-wide association studies (GWAS) has opened for discoveries of new disease-specific genetic variants, targets for drug development, novel molecular signatures, multi-omics integration, improved disease prevention and treatment in a precision medicine perspective.

The HUNT Biobank is a long-term archive facility of biological material, primarily as a biorepository for the HUNT Study. It was established in conjunction with the HUNT2 Survey in 1995–97, and a new biobank facility was opened in 2006 along with the HUNT3 Survey (2006–08). It appears today as an advanced, state-of-the art research biobank (appointed as the European Biobank of the year already in 2013), with automated procedures and instrumentation throughout the entire pipeline from sample handling and storage to retrieval, analysis and delivery of samples. The framework of our quality management system is the Extend Quality System (EQS) and the biobank is certified according to the ISO standard 9001, which ensures that requirements for control of processes and registrations are in place. Data and biological material are stored in agreement with national legislations, ensuring that personal data and material are handled securely. Biological materials from blood, saliva, urine and feces have been collected in line with methods optimal for quality and long-term storage. Standard operation procedures (SOPs) at HUNT adhere to strict national and international regulations and guidelines, all in accordance with the General Data Protection Regulation (GDPR). Best Biobanking Practice for Norwegian Biobanks^6^ and Biobanking for better health care^7^ are implemented in our SOPs. We are at present preparing for an ISO 20387 accreditation,^8^ to further optimize our quality procedures.

## Data collected

All HUNT Study participants were invited to respond to two basic questionnaires, Q1 (100% response rate) and Q2 (75% response rate). Based on various selection criteria, participants may also respond to a number of additional questionnaires, Q3, e.g. diabetes, hypertension, lung disease and cancer (see https://www.ntnu.edu/hunt/data/que).

Self-reported data include socioeconomic position (marital status, education and income), exposures, e.g. CVD medical history, smoking, alcohol, physical activity and dietary assessments.^3^ Basic clinical examinations as well as structured interviews have been conducted by trained personnel. Data from additional add-on studies are also available, e.g. hearing tests, cerebral magnetic resonce imaging, echocardiography and celiac disease.^3^

All the data collected are structured and stored in HUNT Databank, presented in Norwegian and English on the HUNT Databank website (https://hunt-db.medisin.ntnu.no/hunt-db/variablelist). Further information on the data collected may be found in previous cohort profile updates.^1–5^

### Sample collection and handling

Biological material stored at HUNT Biobank has been collected in the non-fasting state at the screening sites from HUNT2 (*n* = 65 228), HUNT3 (*n* = 50 800), HUNT4 (*n *= 56 041), HUNT COVID (*n* = 30 134), Young-HUNT3 (*n* = 8199) and Young-HUNT4 (*n *= 8066). Primary samples collected for further fractionation include whole blood with dimethyl sulphoxide (DMSO) for viable cells, serum-separating tubes (SST)-serum, EDTA-plasma, tempus tubes (RNA), DNA (buffy coat, Oragene saliva kit, buccal swabs), sodium heparin tubes for trace element analyses, tubes without preservatives for both urine and saliva collection and filter paper for fecal sample collection. A complete overview of numbers and types of biological samples in addition to available analyses results are listed in Table 1.

**Table 1.** Overview of type and number of stored biological material from the various HUNT (the Trøndelag Health Study) surveys, with information on selected analytical data and measurements performed

	Survey						
	HUNT1	HUNT2	HUNT3	HUNT4	Young-HUNT1	Young-HUNT3	Young-HUNT4
Years of collection	1984–86	1995–97	2006–08	2017–19	1995–97	2006–08	2017–19
Age group (years)	19–110	19–110	19–110	19–110	13–19	13–19	13–19
Invited	86 404	93 898	93 860	103 799	10 202	10 464	10 608
Participated^a^ (%)	77 202 (89)	65 228 (69)	50 800 (54)	56 041 (54)	8980 (88)	8199 (78)	8066 (76)
Biological samples							
Extracted DNA	—	62 585	50 057	53 309	—	7632^b^	6719^c^
Genotyped (Illumina-Human Core Exome)^12^^,^^13^^,^^21^^,^^22^	46049 ^d^	58 569	50 115 ^d^	54 066 ^d^	3400 ^d^	3045^d^	264
Methylation (HumanMethylation450 Bead Chips, Illumina 485 000 CpG sites)^23^^,^^24^		785	146	9			
Serum	491	64 058	49 492	54 002	—	—	—
Metabolomics^25–29^							
NMR, Nightingale		672	19 921				
Metabolon			234				
Biocrates AbsoluteIDQ p180 kit		488	174				
NMR spectroscopy, lipidomics and metabolites^28^		2311	218				
miRNA^e^^30^^,^^31^		720	442				
Celiac disease markers (anti-TG2 IgA and IgG)^32^				54 541			
Troponin I^33^		9711	5484	37 825			
Plasma	—	—	49 050	52 529	—	—	—
Proteomics^34–37^							
Somalogic SomaScan 1000			1017				
Somalogic SomaScan 5000			3271				
Somalogic SomaScan 7000		42	2227				
Olink Target 96-panel^f^		162	2143				
Feces (smear on paper)	—	—	—	13 257	—	—	—
Microbiome DNA				13 257			
Microbiome profiling (PMP, Bio-Me)^38^				5667			
Shotgun metagenomic sequencing				13 257			
Urine^39^^,^^40^	—	—	16 475	27 160	—	—	—
First morning urine			5759				
Spot urine			11 984	27 160			
Metabolomics							
Biocrates Absolute IDQ p180 kit^41^			312				
Full blood	—	—	26 869	—	—	—	—
Trace element analysis^42^^,^^43^			2266				
Full blood for creation of immortalized cells (ACD, Becton, Dickinson)	—	—	40 734	—	—	—	–
Full blood for total RNA (Tempus-tubes, Thermo Fisher Scientific)	—	—	14 539	—	—	—	–
RNA isolated			328				
Saliva	—	—	—	17 509	—	—	

IDQ, ID Quantique; NMR, nuclear magnetic resonance spectroscopy; PMP, Precision Microbiome Profiling (PMP™) platform by Bio-Me; ACD, acid citrate dextrose.

a Participants with questionnaire data.

b DNA fixed on FTA paper (Flinders Technology, a technology trademarked and patented by Whatman, GE Healthcare Life Sciences, UK).

c DNA from saliva (Oragene OG-500, DNA Genotek).

d Due to the longitudinal study design (see Supplementary Figure S1, available as Supplementary data at *IJE* online) many participants gave samples at several HUNT surveys. Hence, numbers do not necessarily agree with the survey-specific numbers.

e Small RNA Sequencing (Illumina), Serum Focus miR PCR Panels (Exiqon, Vedbaek, Denmark) and miR Pic-and-mix System (Exiqon, Vedbaek, Denmark).

f Olink Cardiometbolomic, Olink Cardiovascular II, Olink Cardiovascular III, Olink Development, Olink Immuneresponse, Olink Metabolism, Olink Oncology II, Olink Organ damage (Olink, Sweden; https://www.olink.com/).

**Figure 1.**
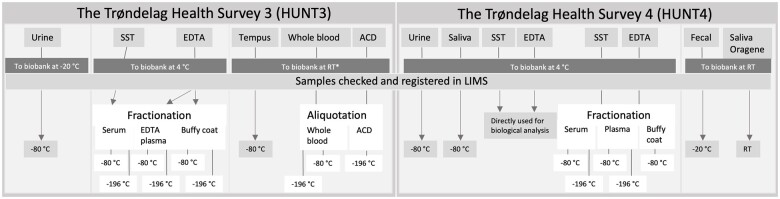
Sample handling in the Trøndelag Health Survey 3 (HUNT3; 2006–08) and the Trøndelag Health Survey 4 (HUNT4; 2017–19) at screening sites and HUNT Biobank. SST, serum-separation tube with clot gel; EDTA, ethylene diamine tetra-acetic acid; ACD, acid citrate dextrose; LIMS, Laboratory Information Management System; RT, room temperature

Details of samples, tubes and sample-handling procedures at screening sites and during transportation, are listed in Table 2 and Supplementary Material (available as Supplementary data at *IJE* online). The Biobank sample handling and storage procedures are illustrated in Figure 1.

**Table 2.** An overview of sample types in surveys of the Trøndelag Health Study (HUNT2, HUNT3 and HUNT4); tubes for purpose, supplier and processing at screening sites and during transport to the biobank

Type of sample	Survey	Vacutainer tube	Supplier	Sample condition at arrival in biobank	**Temperature at field station and during transport to biobank** ^a^
SST	HUNT2	7.5 ml	Becton, Dickinson (BD), NJ	Centrifuged	(2–8 °C)^b^
	HUNT3	8.5 ml	Becton, Dickinson and Company (BD), New Jersey, USA	Centrifuged	(2–8 °C)^b^
	HUNT4	8 ml	Greiner Bio-one, Austria	Centrifuged	(2–8 °C)^b^
	HUNT4	2.5 ml	Greiner Bio-one, Austria	Centrifuged	(2–8 °C)^b^
EDTA	HUNT2	5 ml	Becton, Dickinson, (BD), NJ	Non-centrifuged	(2–8 °C)^b^
	HUNT3	10 ml	Becton, Dickinson (BD), NJ	Non-centrifuged	(2–8 °C)^b^
	HUNT4	9 ml	Greiner Bio-one, Austria	Non-centrifuged	(2–8 °C)^b^
	HUNT4	3 ml	Greiner Bio-one, Austria	Non-centrifuged	(2–8 °C)^b^
ACD	HUNT3	5 ml	Becton, Dickinson (BD), NJ	Non-centrifuged	RT in a Styrofoam box
Whole blood containing sodium heparin or EDTA	HUNT3	5 ml	Becton, Dickinson (BD), NJ	Non-centrifuged	RT in a Styrofoam box
RNA, whole blood in Tempus tubes	HUNT3	9 ml	Thermo Fisher Scientific, Waltham, USA	Non-centrifuged	RT in a Styrofoam box
Spot urine, Urine collection kit	HUNT3 HUNT4	9.5 ml	Greiner Bio-one, Austria	Non-centrifuged	Immediately frozen at –20 °C
Morning urine	HUNT3	5 ml	Cryotubes, Nunc, Thermo Fisher Scientific, UK	Non-centrifuged	RT (by post)
Saliva, screw cap tube	HUNT4	15 ml	Sarstedt, Germany	Non-centrifuged	(2–8 °C)^c^
Fecal samples, FTA cards (Whatman 903^®^ filter paper)	HUNT4	—	Whatman, GE Healthcare Pittsburg, USA	—	RT (by post)
Buccal smears FTA cards (Whatman filter paper)	Young-HUNT3	—	Whatman, GE Healthcare Life Sciences, UK	—	RT
Saliva, Oragene OG-500 kits	Young-HUNT4		DNA Genotek Inc, Ontario, Canada	Non-centrifuged	RT

SST, serum separation tube with clot gel; EDTA, ethylene diamine tetra-acetic acid; ACD, acid citrate dextrose; RT, room temperature; FTA, Flinders Technology, a technology trademarked and patented by Whatman.

a All samples stored at HUNT (the Trøndelag Health Study) Biobank are under continuous temperature alarm-based monitoring.

b Logged temperature monitoring in refrigerator and transport cooler.

### Laboratory Information Management System (LIMS)

To ensure optimal management and tracking within our biological sample repository, HUNT Biobank has developed its own Laboratory Information Management System (LIMS), the HUNT Biobank Resource Information System—Hubris. It is based on an open-source relational database management system (Dataphor), with associated user interface technologies, well suited to in-house development of database applications.

More specifically, Dataphor allows for automated and safe handling of large datasets and for arrays of associated data and statistics to be linked to the samples. In HUNT Biobank, it is used for sample monitoring, project delivery and inventory management of information linked to each sample such as remaining volume, concentration, analysis results and quality. The system is hosted in a closed and local network environment with no internet connection, which ensures that access to data is strictly regulated and limited to what is specifically needed for each research project. Participants’ PIN is required for linkage to registries and other data sources, but only a few dedicated personnel have access to this information.

### Storage facility

Fit-for-purpose automated storage solutions for biological material are implemented at HUNT Biobank, where the biological material is stored at temperatures optimal for sample type and purpose, ranging from storage temperatures at room temperature to -196°C; see Table 3 for more detailed information.

**Table 3.** Overview of HUNT^b^ Biobank’s storage facilities

Storage facility	Temperature	Supplier	Capacity	Biological specimens
Liquid nitrogen freezers (gas phase)	–196 °C	Statebourne Cryogenics, UK	16 containers with 100 000 Matrix tubes^a^	ACD blood/plasma/serum/urine/whole blood
Chest freezers: Forma –86C ULT freezer	–80 °C	Thermo Scientific, USA	38 freezers with 65 000 Matrix tubes^a^	Buffy coat/DNA/plasma/RNA/serum
Automatic BioStore II	–80 °C	Azenta Life Sciences, UK	∼6 million Matrix tubes^a^	Buffy coat/DNA/plasma/serum/urine/whole blood
Chest freezers	–20 °C	Frigor, Italy	16 freezers with 50 000 Matrix tubes^a^	DNA/fecal samples
Fireproof cabinets	Room temperature	Seifuva, Lithuania	—	Buccal cells (FTA card)

ACD, acid citrate dextrose; ULT, ultra-low temperature; FTA, Flinders Technology Associates, a technology trademarked and patented by Whatman.

a 1.4-ml matrix tubes.

b HUNT, the Trøndelag Health Study.

In addition to processing and storage of samples collected in The HUNT Study, HUNT Biobank offers long-term storage and analyses of biological material from a number of external collections, e.g. a national DNA-sample collection representing all major population studies in Norway (CONOR, Cohort of Norway).^9^

### DNA and RNA extractions, quality assurance and biomarker analyses

Since the initial sample collections in 1995–97 (HUNT2), there has been a continuous knowledge-based method improvement within the biobanking field; including the transition from manual to automatic sample handling procedures. The concentration and purity of DNA samples have been routinely monitored to ensure continuous good quality in our samples. DNA yield is expected to vary depending on quality and number of cells in each blood sample, from 8 µg to 50 µg DNA per ml.^10^ The average DNA yields from HUNT whole-blood samples is 20 µg/ml (SD = 10 µg/ml) after long-term storage (i.e. >10 years of storage at -80°C), which is fully acceptable. DNA extraction procedures and quality assurance measures are described in more detail in Supplementary Table S1 and Supplementary Material (available as Supplementary data at *IJE* online).

To ensure measurement accuracy and high quality of DNA samples, HUNT Biobank has participated in both the International Society for Biological and Environmental Repositories (ISBER) and Integrated Biobank of Luxembourg (IBBL) annual proficiency-testing programmes since 2012 and 2014 (DNA quantification and purity testing programmes), respectively, to warrant optimal sample quality.

Genome-wide genotyping has been performed using an Illumina custom-made Human Core Exome chip array (Sequenom, San Diego) consisting of direct genotyping of 358 964 polymorphic variants.^11^ Further imputation procedures have resulted in a total of 33 million variants in 88 517 individuals from HUNT2, HUNT3 and HUNT4.^11^

For projects involving only a few single nucleotide polymorphisms (SNPs) related to specific phenotypes, genotyping is performed at HUNT Biobank with a throughput of ∼5000 samples targeting single SNPs analyses per day (instruments listed in Supplementary Table S2, available as Supplementary data at *IJE* online).

Biochemical and immunological analyses of serum, plasma and urine are carried out using clinical biochemical or immunochemical approaches, instruments listed in Supplementary Table S2 (available as Supplementary data at *IJE* online). For in-house biomarker analyses, see examples listed in Table 4.

**Table 4.** In-house biomarker analyses performed at HUNT Biobank and type of instrument used for the analyses

Type of biomarker	Number of samples	Biological material	Instrument
Total vitamin D (25OH)^30–34^	18 500	Serum	Liaison
Creatinine	7300 + 30 000	Urine, serum	ABX Pentra 400, Pentra C400
Microalbumin	7300	Urine	ABX Pentra 400
Cystatin C	4700	Serum	ABX Pentra 400
C-reactive protein (CRP)	30 000	Serum	Pentra C400
Albumin	40 000	Serum	ABX Pentra 400, Pentra C400
Serological marker for *Helicobacter pylori* (ELISA)	5800	Serum	Tecan Freedom Evo 150

ELISA, enzyme-linked immunosorbent assay.

### Sample delivery

HUNT Biobank has been an attractive and frequently used supplier of biological materials for a wide range of research projects over the past 30 years. We handle and deliver biological material for projects collected through all the HUNT surveys, but also serve external projects by managing their sample collections. Delivery of biological material from HUNT is primarily in the format of matrix tubes or 96-well plates. Major sample deliveries are listed in Table 1.

## Data resource use

The comprehensive phenotypic and genetic data available from the HUNT cohort have resulted in new discoveries of genetic associations and candidate variants across a broad range of traits, e.g. atrial fibrillation.^12^ In a study by Holmen *et al*.,^13^ a novel coding low-frequency variant in the TM6SF2 (p.Glu 167Lys) gene with functional follow-up in mice was shown to affect total cholesterol levels, and reduced the risk of myocardial infarction. In this study, HUNT was used as a discovery cohort (*n* = 5771) and findings were replicated in another Norwegian cohort, the Tromsø Study (*n* = 4666).^14^ The high citation rate on this publication, the successful replication of findings and further verification of functional characteristics confirm the high quality and importance of the genetic data that originate from HUNT.

The HUNT Study has made significant contributions to many international collaborative genome-wide studies, e.g. the GIANT (Genetic Investigation of Anthropometric Traits) consortium.^15^ Studies based on the GIANT consortium have had a great impact identifying loci associated to anthropometric traits,^16^^,^^17^ which has further enabled the disentangling of complex interactions between genetic predisposition and environment for traits such as obesity.^18^ For further examples of the use of biological material and data from HUNT, see publications listed in Table 1.

To avoid duplication of efforts by different research groups, bias due to variability in methods and instrument variability, and batch effects, several large-scale omics studies have been initiated and organized by HUNT, including genome-wide genotyping (88 517), metabolomics (17 000), proteomics (5700) and metagenomic sequencing of 12 800 fecal samples. These are resources available for the wider research community.

## Strengths and weaknesses

Major strengths of our data resource are: structural recruitment from a large, longitudinal, population-based cohort, with repeated measurements over almost four decades; a strong emphasis on high-quality sample handling and management; and the vast amount of self-reported health data through structural questionnaires, interviews and clinical examinations. Incident clinical outcomes are harvested and validated through linkage to regional and national registries, based on a unique personal identification number (PIN). This is a resource highly requested by the national and international research communities and an excellent resource for biomarker validation and risk prediction. Further surveys of the HUNT Study are in the planning, with HUNT5 starting in 2027; hence the number of samples, repeated measures and new health data will continuously increase. The large number of families included makes intergenerational studies possible, where genetic data and biological samples are available for studies of trios and within-family mendelian randomization.^19^

In HUNT2 (1995–97), a limited amount of serum was collected compared with the subsequent surveys (HUNT3 and HUNT4), and DNA was extracted from blood clots and not from EDTA-tubes for half of the samples, introducing some degree of fragmentation. Consequently, there is some variability in DNA yield and volumes in samples from this survey compared with the following surveys. However, this has not had any impact on the subsequent genetic analyses.

We have not collected blood from the Young-HUNT participants in case this could affect their motivation to participate in the studies. However, less invasive sampling procedures involving buccal smears and saliva samples for DNA analyses were collected in Young-HUNT3 (2006–08) and Young-HUNT4 (2017–19), respectively. Even so, 6000 Young-HUNT participants have later taken part in HUNT3 and HUN4 as adults, and thereby contributed with biological material for genome-wide profiles.

Fecal samples were collected from 13 257 participants in the HUNT4 Survey.^20^ Even though this includes only 25% of the HUNT4 participants, 50% have participated in all four HUNT surveys, and it is one of the largest population-based fecal sample collections internationally, already proven to be a very valuable and attractive scientific resource.^20^

## Data resource access

Data, biological material and analysis services offered by HUNT Biobank are accessible upon request to HUNT Research Centre (https://www.ntnu.no/hunt/) provided that approval from the Regional Committee for Medical and Health Ethics (REC) is in place. Data can be made available for researchers affiliated to an approved research institution; however, international research groups, including industrial-based research initiatives, must collaborate with and apply through a Norwegian principal investigator.

Information about biological material available for analysis and analysis services offered by HUNT Biobank can be found on HUNT Biobanks website (https://www.ntnu.no/hunt/biobank). A detailed overview of available data is provided on HUNT Databanks website (https://www.ntnu.no/hunt/databank). Sharing of HUNT data only takes place in accordance with the GDPR. Large datasets, e.g. GWAS data and other omics data, may be accessed via HUNT Cloud (https://www.ntnu.edu/mh/huntcloud), complying with two international standards, IEC/ISO 27001 for information security and privacy and IEC/ISO 9001 for quality management. For more information about HUNT, you may visit our website (http://www.ntnu.no/hunt/) or contact Elin Pettersen Sørgjerd (kontakt@hunt.ntnu.no).

## Ethics approval

More than 140 000 participants from The HUNT Study have actively consented to biobank-based research and the use of registry-based linkage to clinical endpoints/phenotypes. Population studies were previously subject to approval by the Mid-Norway Regional Committee for Medical and Health Research Ethics and the Data Inspectorate. Since 2019, population-based data and sample collections are regulated by the National Regulations on Population-Based Health Surveys, Norway.

## Supplementary Material

dyae073_Supplementary_Data

## Data Availability

See Data Resource Access, above.
