# Supplementary material for: Data Resource Profile: The HUNT Biobank
Source: Int J Epidemiol. 2024 Jun 4;53(3):dyae073. doi: 10.1093/ije/dyae073 (PMC11150882; doi:10.1093/ije/dyae073)
Supplement: dyae073_Supplementary_Data [file dyae073_supplementary_data.docx]

**Supplementary material.**

**Abbreviations:**

HUNT The Trøndelag Health Study

ACD Acid Citrate Dextrose

SST Serum Separating Tube with clot gel

EDTA Ethylene Diamine Tetra acetic Acid

PCR Polymerase Chain Reaction

**Sample collection and handling**

**HUNT2: Samples** were collected between August 1995 and June 1996. Blood drawn in SST tubes (sample serum separation tube) were left for 30-90 minutes at room temperature before centrifugation, placement, and further transport at 4 °C to HUNT Biobank. From August 1996 to June 1997, an additional EDTA (Ethylene Diamine Tetra acetic Acid) blood tube was collected from each participant.

The same day or within 2-3 days (when over weekends) of collecting the SST-sample, biomarker measurements were performed at Levanger Hospital (next-door to HUNT Biobank). The remaining serum (approximately 1.5 ml per sample) was stored in cryotubes at -70 °C or later aliquoted to be stored at -80 °C. Clots from centrifuged SST tubes or whole blood from EDTA-tubes were stored at -20 °C and -70 °C respectively, pending later DNA extraction.

**HUNT3:** Samples were collected between October 2006 and June 2008. SST tubes were handled similarly as in HUNT2, while EDTA tubes from all participants were preserved at 4 °C immediately after sampling, but not centrifuged until the samples arrived the biobank. Additionally, an ACD (Acid Citrate Dextrose) blood tube for potential establishment of immortalized cell lines was collected from most of the participants (n= 40 700). Further, one tube of whole blood for trace elements analysis containing the anticoagulants Sodium Heparin (n= 24 420) or EDTA (n= 1605) was collected from the first period of the sample collection. This tube was skipped halfway through the total sample collection period and replaced by a Tempus blood RNA tube for a sub-sample (n= 14 800). Spot urine samples, frozen at -20 °C within 30 minutes, were collected in a Vacutainer Urine Collection Kit from a sub-sample of participants (n= 11 944). The collected samples were temperature-monitored at the screening sites and during the transport to HUNT biobank in the evening. At the biobank SST tubes, EDTA and Tempus RNA samples were placed at 4 °C (logged and monitored), while the ACD and whole blood samples containing anticoagulant (Sodium Heparin or EDTA) were kept in room temperature until next morning for further processing.

Next morning, EDTA samples were centrifuged (RCF = 2500g, 15 minutes, 6 °C) and both the EDTA and SST samples were fractionated and dispersed into 2D Matrix tubes (Matrix™ 2D, ThermoFisher, USA). Fractionation was performed below 10 °C using the RTS Blood Fractionation System (RTS Life Science, Manchester, UK). The ACD and whole blood samples containing anticoagulant, were aliquoted into 2D Matrix tubes (room temperature) using Tecan Freedom EVO (Tecan, Switzerland). Processed SST, EDTA and whole blood aliquots were immediately frozen at –80 °C (see Figure 1 in the main text). 20 % sterile cryoprotective agent (DMSO) in the ratio 1:1 to was added to the aliquoted ACD samples before further transferal to –196 °C LN2 using a slow cooling procedure (~1 °C decrease per minute).

Applicable to the samples was that they were all processed and stored at HUNT biobank within 36 hours from the time of sampling at the screening sites.

A selection of participants (n= 5700) also submitted three morning urine samples. Selection criteria were based on either 1) participation in the *Micro Albumin Project* in HUNT2, 2) reporting having diabetes in HUNT3, or 3) were within the randomly selected 10% general HUNT3 participant. These samples were sent to HUNT biobank by post.

**HUNT4:** Samples were collected between September 2017 and June 2019. Same procedures as in HUNT3 were used for collecting SST- and EDTA blood samples. SST samples were centrifuged (RCF = 2200g, 10 minutes) within 2 hours from phlebotomy before being placed at 4 °C, while EDTA samples followed the same procedures as in HUNT3 (Table 2 in the main text).

From randomly selected 50% HUNT4 participants, spot urine samples were collected (Vacutainer Urine Collection Kit) and placed in a refrigerator within two hours of collection with continuous temperature logging (2 – 8 °C). Saliva samples were collected at random where participants were asked to passively drool continuously for three minutes into a 15 ml tube, which afterwards were placed directly in the refrigerator. At the end of the day, all samples were transported to the biobank in transport boxes (temperature 2 - 8 °C, logged and monitored). The samples were stored at 4 °C until the next morning, before further processing within 36 hours after phlebotomy.

At biobank arrival, the EDTA samples were centrifuged (RCF = 2500g, 15 minutes, 6 °C), and further fractionated and dispersed into 2D Matrix tubes using Tecan Freedom EVO 200 (Tecan, Switzerland). This was done at room temperature due to new optimized sample handling procedures since HUNT3. Centrifuged SST samples were handled as for the EDTA samples, alternatively by using Tecan Freedom EVO 150. Samples were aliquoted and immediately placed at -80 °C.

Fecal samples (n= 13 296) were collected on FTA cards by participants at home following instructive written procedure information, where after the cards were sent to the biobank by post.

**Young-HUNT3:** For DNA, buccal smear samples were collected, and each participant followed the procedures according to the manufacturers’ written instructions. Samples were fixed directly on FTA-cards, which were further stored at room temperature in a fireproof cabinet.

**Young-HUNT4:** For DNA, saliva samples were collected using Oragene OG-500 kits and stored at room temperature until DNA extraction, all according to the manufacturer’s procedures.

**DNA and RNA extractions, quality assurance and biomarker analyses**

NanoDrop or DropSense 96 Trinean, were used to routinely test concentration and purity in every eighth DNA sample (spectrophotometric measurements, A260/A280) (Table S2). Fresh samples are expected to give highest yields and our measurements are mostly based on long-term stored samples (- 80 °C). An A260/A280 ratio of 1.8 is generally accepted as pure for DNA [1], which is the average ratio found in our samples with a standard deviation of 0.2.
In all DNA samples, Biomek NX (2006 – 2017) and Biomek i5s8 (2017 until present) with PicoGreen dsDNA quantification (Invitrogen, USA) were used for concentration measurements prior to project delivery. Sample specific dilutions, i.e., defined volumes and concentrations were made either based on project specific requirements or concentrations of the original samples. Predefined concentrations of 50 ng/µl were found to be 50.19 SD ± 9.9 ng/µl after dilution procedures. Further, for measuring DNA fragmentation defined as DNA integrity (DIN), the Agilent TapeStation 4200 was used for in-house validation of DNA-methods and sometimes on requests from project owners. In the ISBER and IBBL proficiency-testing programs that HUNT Biobank took part in, the DNA concentration was measured using Qubit and Biomek i5s8 connected to FilterMax F5, Multi-Mode Microplate Reader, while DNA purity (Ratio A260/A280) and spectrophotometric concentration were measured using NanoDrop.

The Tempus blood RNA tubes, used in HUNT3, was designed for collection and stabilization of RNA for gene expression analyses. From this resource, RNAs are extracted either by the automated procedure using the Maxwell® 16 LEV simply RNA Blood Kit and the Maxwell® 16 Instrument or by using the Chemagic 360. Further information about the instruments is to be found in Table S2.

1. Inc TFS. NanoDrop 1000 Spectrophotometer v.3.8 User`s Manual web page: Thermo Fisher; 2010 [2022 27.04]. Manual]. <https://tools.thermofisher.com/content/sfs/manuals/nd-1000-v3.8-users-manual-8%205x11.pdf>. (27. April 2022, 14. April 2024).

**Table S1**. **Overview of DNA extraction methods in The Trøndelag Health Study (HUNT)*.***

| **Survey** | **DNA source** | **Extraction kit** | **Protocol** | **Supplier** |
| --- | --- | --- | --- | --- |
| HUNT2 | Peripheral blood leukocytes from EDTA full blood or blood clots | Gentra Purgene Blood kit | Manually or  Automated (Autopure LS) | QIAGEN Science, Maryland, USA |
| HUNT3 | Buffy coat (EDTA) | MasterPure DNA purification Kit | Manually | Epicentre Biotechnologies, USA |
|  |  |  | Automated (Chemagic Star, by Hamilton, Switzerland) | Perkin Elmer, Germany |
| HUNT4 | Buffy coat (EDTA) | Chemagic^TM^ reagents kit | Automated (Chemagic Star, by Hamilton, Switzerland) | Perkin Elmer, Germany |

EDTA, Ethylene Diamine Tetra acetic Acid

**Table S2. Use of instruments for in-house analyses per 2022 at HUNT Biobank.**

| **Instrument** | **Supplier** | **Area** |
| --- | --- | --- |
| ABX Pentra 400 | Bergman Diagnostika / Horiba, France | Biochemical analysis |
| Pentra C400 | Bergman Diagnostika / Horiba, France |  |
| Liaison XS | Diasorin, Italy | Immunochemical analysis |
| NanoDrop One | BioNordika /Thermo Fisher, USA | Spectrophotometry analysis of DNA quantity and quality |
| DropSense96 Trinean | Hamilton / Techtum, Sweden |  |
| Biomek i5s8 | Nerliens Meszansky AS / Beckman Coulter Life Sciences, USA | Fluorescence analysis DNA quantity |
| Qubit | ThermoFisher Scientific, USA |  |
| QuantStudio 5 | Thermo Fischer Scientific, USA | PCR / SNP genotyping |
| Aglient TapeStation 4200 | Matriks AS / Agilent Technologies, USA | DNA and RNA Integrity analysis  (RIN, DIN) |
| Tecan Freedom Evo 150 | Bergman Diagnostika / Tecan, Switzerland | Fractionation/aliquotation |
| Tecan Freedom Evo 200 | Bergman Diagnostika / Tecan, Switzerland |  |
| Biomek i5MC | Nerliens Meszansky AS / Beckman Coulter Life Sciences, USA | Dilution and transfer |
| Hamilton Chemagic Star | Hamilton, Switzerland | Extraction of DNA |
|  | PerkinElmer, USA |  |
| Chemagic 360 | PerkinElmer, USA |  |
| Maxwell® 16 | Nerliens Meszansky AS / Promega, USA | Extraction of RNA  (including miRNA) |
| Chemagic 360 | PerkinElmer, USA |  |

PCR, Polymerase Chain Reaction; SNP, Single-nucleotide polymorphism; DNA, Deoxyribonucleic acid; RNA, Ribonucleic acid; miRNA, microRNA

**
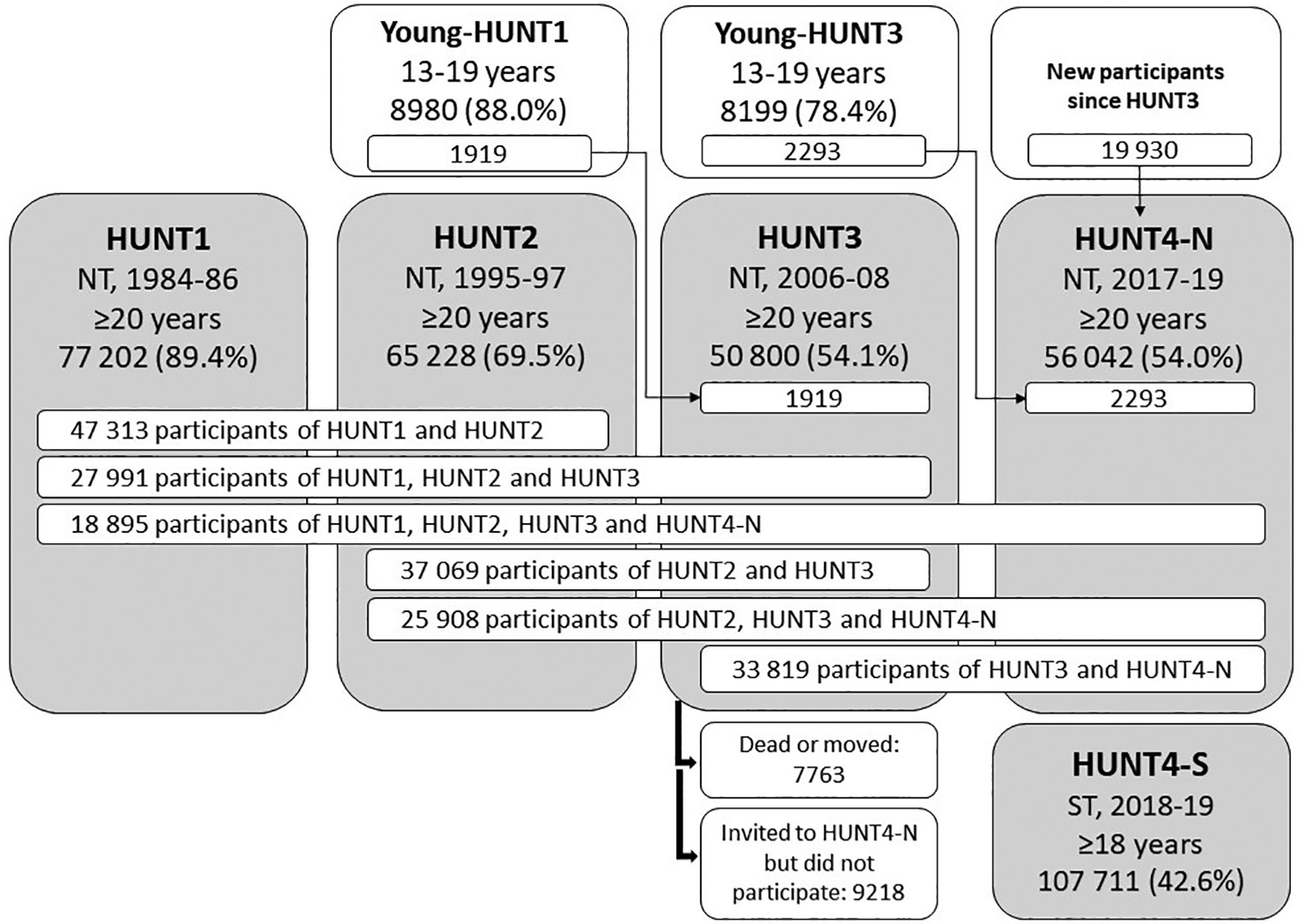
**

**Figure S1:** Flowchart of participation across The Trøndelag Health Study, during the HUNT1 – HUNT4 surveys.
Originally published in the Cohort Profile Update: The HUNT Study, Norway [2].

2. Åsvold BO, Langhammer A, Rehn TA, et al. Cohort Profile Update: The HUNT Study, Norway. International journal of epidemiology. 2023;52(1):e80-e91.
